# Construction and Characterization of Novel Hydrophilic Nanospheres Loaded with Lambda-Cyhalothrin via Ultrasonic Emulsification–Solvent Evaporation

**DOI:** 10.3390/ijms232214063

**Published:** 2022-11-15

**Authors:** Chunxin Wang, Mengjie Wang, Yan Wang, Junqian Pan, Changjiao Sun, Zhanghua Zeng, Shuaikai Ren, Haixin Cui, Xiang Zhao

**Affiliations:** Institute of Environment and Sustainable Development in Agriculture, Chinese Academy of Agricultural Sciences, Beijing 100081, China

**Keywords:** lambda-cyhalothrin, nanospheres, hydrophilicity, leaf adhesion, ultrasonic emulsification, sustained-release

## Abstract

Safe and efficient pesticide formulations have attracted great attention for the prevention and control of diseases and pests. In recent years, improving the effectiveness and duration of pesticides through nanotechnology has become a research hotspot in the field of pesticide formulations. Here, we develop a novel hydrophilic lambda-cyhalothrin nanospheres encapsulated with poly(styrene-co-maleic anhydride) (PSMA) via the ultrasonic emulsification–solvent evaporation method, which exhibited better particle size uniformity and dispersion in comparison with the traditional method. The effects of PSMA content, oil phase/water phase ratio and phacoemulsification time on the particle size and morphology of nanoparticles were investigated to optimize preparation process parameters. Meanwhile, the wettability and adhesion behavior on the leaf surface, the release properties, and the storage stability of nanoparticles were characterized to evaluate the performance of the novel nano-formulation. This work not only establishes a facile and promising method for the applicable of insoluble pesticides, but also develops an innovative nano-formulation with hydrophilicity and high leaf adhesion, which opens a new direction in plant protection and residue reduction.

## 1. Introduction

Pesticides are considered the most effective method to control diseases and pests. Therefore, improving the effective utilization of pesticides has become the focus of pesticide formulation research [1,2,3]. The dissolution rate of pesticides is directly in proportion to the surface area of particles according to the Noyes–Whitney equation [4]. Therefore, reducing the particle size and then increasing the specific surface area of pesticide particles have become urgent problems to be solved. Pesticide particles were processed by nanotechnology to prepare nano-formulations, which can significantly improve the effective utilization of pesticides [5,6,7,8]. As we all know, nanotechnology has great research and application prospects in agriculture, and its achievements are also widely used in the field of pesticides. Zhao reported that the development and application of new nanopesticide formulations are of great significance to promote the research of pesticide formulations [9]. Wang reported that the lambda-cyhalothrin nanoparticles with tunable particle size have better physicochemical properties and biological activities, which can act on insect targets more efficiently [10].

As one of the representatives of pyrethroid insecticides, lambda-cyhalothrin has the characteristics of wide insecticidal spectrum and rapid efficacy. It can inhibit the conduction of insect nerve axons and has toxic effects on pests. However, the poor water solubility and low target efficiency of lambda-cyhalothrin limit its insecticidal application in farmland systems. Therefore, it is urgent to develop a pesticide formulation with good water solubility and high leaf adhesion and deposition rate to improve target pest exposure time. Nano-formulations can promote the dispersibility of active ingredients in water and improve the deposition rate in the target [11,12]. Keeping the particle size distribution uniform and avoiding agglomeration between particles has become the focus of nano-formulation research. It has been proven that the size and surface properties of nanoparticles can affect the interaction between pesticide particle and crops target [13,14,15,16]. Therefore, controlling the particle size of nanoparticles and overcoming the agglomeration between particles have become the focus of current formulation research. As a functional polymer, poly(styrene-co-maleic anhydride) (PSMA), with a wide source and low price, was mainly used as drug carrier material. PSMA contains hydrophobic styrene groups and hydrophilic anhydride groups, which are amphiphilic. Moreover, it can be used to adjust the size and surface potential of polymer nanoparticles and prevent the aggregation of nanoparticles at high concentrations [17,18,19,20].

**Scheme 1:**
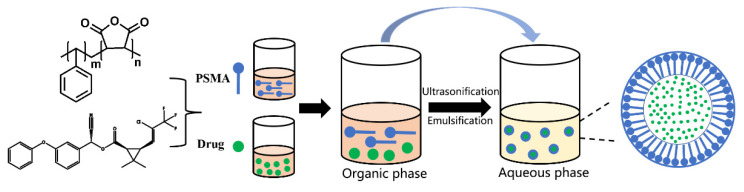
Schematic representations of construction of lambda-cyhalothrin nanospheres.

In this study, the hydrophilic lambda-cyhalothrin nanospheres with PSMA as carrier were prepared using the ultrasonic emulsification–solvent evaporation method [21,22,23,24] (Scheme 1). The effects of PMSA content, oil–water ratio, and ultrasonic emulsification time on the particle size of nanoparticles were estimated, and the preparation process was optimized. The effects of surface structure and particle size of the nanospheres on the wettability, leaf adhesion, release properties, and stability of nanoparticles were explored. This research not only provides a simple and quantitative preparation technology for insoluble pesticides, but also lays a foundation for the study of the nano-formulation with hydrophilicity and high leaf adhesion.

## 2. Results and Discussion

### 2.1. Characterization of the Nanospheres

**Figure 1:**
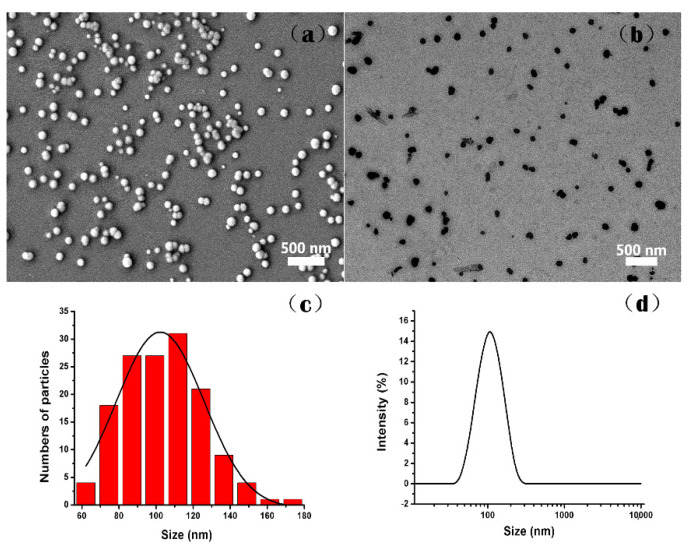
Characterization of particle size and morphology of the PSMA-LC-NS. (**a**) SEM image; (**b**) TEM image; (**c**) statistical particle size based on SEM image; (**d**) DLS image.

Lambda-cyhalothrin nanospheres encapsulated with PSMA (PSMA-LC-NS) were prepared using the ultrasonic emulsification–solvent evaporation method in the fabrication process. As shown in Table 1, the mean particle sizes of the PSMA-LC-NS and lambda-cyhalothrin particles were 105 ± 0.6 nm and 248 ± 5.6 nm, respectively. The mean particle size of PSMA-LC-NS based on scanning electron microscope (SEM) image (Figure 1a) and transmission electron microscope (TEM) image (Figure 1b) were 103 nm and 102 nm, respectively, which was smaller than the hydrated particle size (Figure 1d). The main reason was that the nanoparticles are the real particle size in a dried state in SEM and TEM image, whereas the nanoparticles are composed of micelle core and swollen corona in the dynamic light scattering (DLS) test [25,26,27]. Moreover, the prepared PSMA-LC-NS exhibited almost spherical morphology and comparatively monodisperse distribution.

As known to us, zeta potential around ±30 mV means that the nano-system is stable due to the combined electrostatic action and steric effect. The higher the absolute value (positive or negative) of zeta potential is, the more stable the system will be [28]. The zeta potential of PSMA-LC-NS was −31.5, indicating that the surface of nanoparticles has negative charge. This is mainly because PSMA can provide charges to increase electrostatic repulsion between particles, which is favorable for the stability of the system.

### 2.2. Optimization of Preparation Parameters

#### 2.2.1. Effect of PSMA Concentration on Nanosphere Morphology

**Figure 2:**
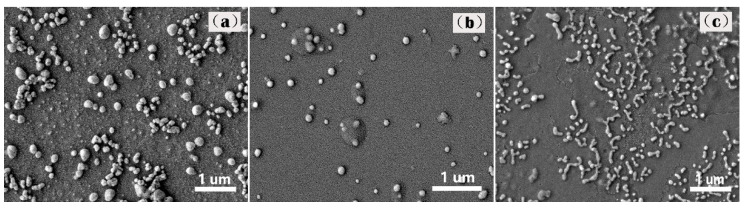
Effect of the ratio of the pesticide/PSMA on nanospheres morphology. Pesticide/PSMA ratios of (**a**) 3:1; (**b**) 1:1; (**c**) 1:3.

The preparation conditions of the PSMA-LC-NS were investigated to optimize the preparation process. The effect of PSMA concentration on the morphology of nanospheres was studied. The ratio of drug/PSMA was set to 3:1, 1:1, and 1:3. The results showed that the PSMA concentration can affect the particle size and uniformity of the prepared nanospheres. As shown in Figure 2b, when the ratio of pesticide/PSMA was 1:1, the particles showed small and uniform particle size compared with that in other ratios of pesticide/PSMA. The main reason may be that the PSMA can completely wrap pesticide particles to form nanoparticles with uniform particle size in order to avoid particle aggregation.

With the increase of pesticide/PSMA ratio, the particle size increases and the particle size distribution is uneven. When the ratio of pesticide/PSMA of the lambda-cyhalothrin particles (LCPs) increased to 3:1, the particle size distribution of the particles was between 100 and 300 nm, and agglomeration occurred easily between the particles (Figure 2a). The main reason may be that the concentration of PSMA is low in the process of particle formation, which cannot be completely combined with the pesticide particles, resulting in the exposure of some particles and the agglomeration between particles. There were adhesion and tailing between particles when the PSMA concentration increased (Figure 2c). The main reason may be that the concentration of PSMA was high, and the excessive PSMA molecules had intermolecular interaction and finally adhered to the particle surface [18]. During the preparation of nanoparticles, too much or too little PSMA would lead to the production of particles with an irregular shape and uneven size. Therefore, the ratio of pesticide/PSMA (1:1) was chosen for the follow-up experiments.

#### 2.2.2. Effect of Volume Ratio of Oil Phase/Water Phase on Nanosphere Morphology

**Figure 3:**
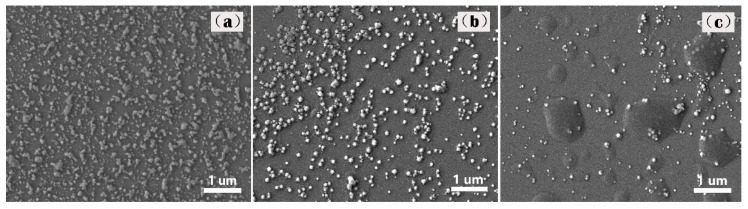
Effect of the volume ratio of the oil phase/water phase on nanospheres morphology. Oil phase/water phase ratios of (**a**) 1:1; (**b**) 1:5; (**c**) 1:10.

As shown in Figure 3, the volume ratio of the oil phase/water phase will affect particle size and the structure of the nanospheres [29]. The results indicated that the prepared nanospheres at the volume ratio of oil phase/water phase of 1:5 have better particle dispersion and uniformity than that of the particles with the volume ratio of oil phase/water phase of 1:10. When the ratio of oil phase/water phase was 1:5, the prepared nanospheres were smooth, and the size uniformity was good.

As shown in Figure 3a, the pesticide particles are prone to agglomerate and adhered to each other in the ratio of oil phase/water phase of 1:1. The main reason may be that the oil-in-water stable system cannot be formed due to the high concentration of oil phase. The content of pesticides and PSMA in the system is also relatively high, which limits the dispersion of the pesticide active ingredient in the aqueous phase and cannot be well dispersed in the aqueous phase. Therefore, the ratio of oil phase/water phase (1:5) was chosen for the preparation of nanospheres.

#### 2.2.3. Effect of Ultrasonic Action Time on Nanosphere Morphology

**Figure 4:**
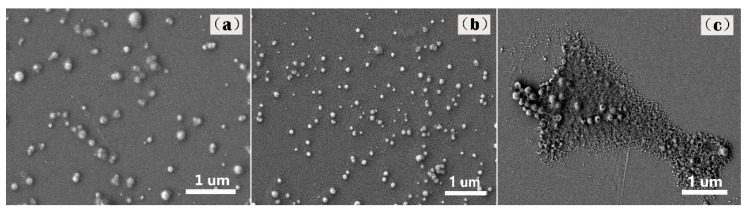
Effect of the ultrasonic action time on nanospheres morphology. Ultrasonic action time of (**a**) 2 min; (**b**) 5 min; (**c**) 10 min.

In order to study the effect of ultrasonic action time on the morphology of nanospheres, the preparation conditions of nanospheres were controlled as follows: the ratio of pesticide/PSMA was 1:1 and the volume ratio of the oil phase/water phase was 1:5. The ultrasonic action time was selected as follows: 2, 5, and 10 min. When the ultrasonic action time is 2 min, the pesticide particles have large particle size and uneven distribution because of the relatively short emulsification and dispersion time (Figure 4a). Therefore, the length of emulsification time directly affects the particle size and morphology. As the ultrasonic action time increased, the size of the prepared particles decreased and the uniformity improved. The prepared nanospheres with the ultrasonic time of 5 min had uniform particle distribution (Figure 4b). However, when the ultrasonic action time increased to 10 min, the surface structure of the nanospheres collapsed, resulting in pores and deformation on the surface (Figure 4c). Nanospheres with appropriate size and morphology can be obtained by controlling the phacoemulsification time. Too long or too short an emulsification time easily leads to particle deformation and adhesion between particles. Therefore, the ultrasonic action time of 5 min was selected.

In conclusion, the optimal preparation conditions for PSMA-LC-NS were as follows: the ratio of pesticide/PSMA was 1:1, the volume ratio of the oil phase/water phase was 1:5, and the ultrasonic action time was 5 min.

### 2.3. Wettability

**Figure 5:**
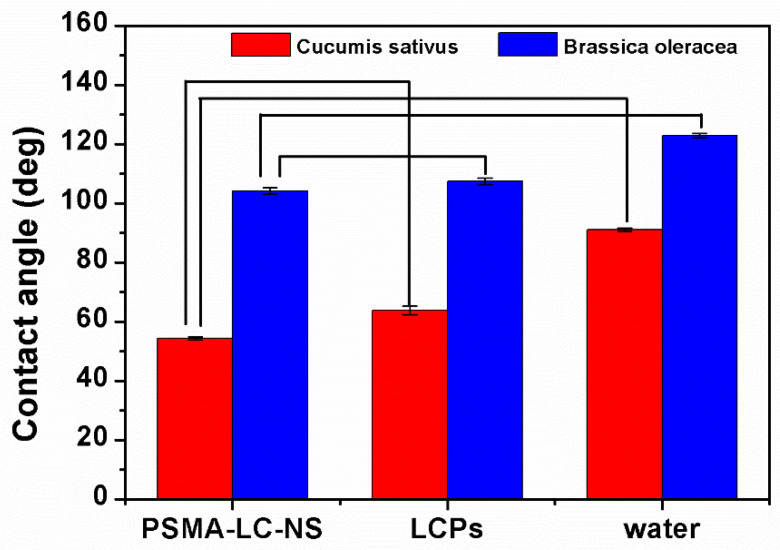
Contact angles of different lambda-cyhalothrin formulations on *Cucumis sativus* and *Brassica oleracea* leaves.

The contact angles on leaves were used to evaluate the wettability of the sample. The LCPs were selected as the control group. The contact angles on *Cucumis sativus* and *Brassica oleracea* leaves were measured and the results are presented in Figure 5. The contact angles of PSMA-LC-NS with the pesticide/PSMA ratios of 1:1 on hydrophilic *Cucumis sativus* leaves was 54.4° ± 0.5°, which was smaller than that of the LCPs. The PSMA on the surface of nanoparticles has hydrophilic groups and can bind to the leaf surface, and enhance the solubility of pesticides and improve the wetting effect. Similarly, the contact angles of PSMA-LC-NS on hydrophobic *Brassica oleracea* leaves was 104.2° ± 1.1°, which was smaller than that of the LCPs. The smaller the contact angle of the sample on the crop leaf surface, the better the wettability of the sample. The results indicated that the nanoparticles have better wettability on the leaf surface, which might be attributed to the reduction of surface tension [30].

The main reason for the excellent wettability of the PSMA-LC-NS was probably that PSMA molecules wrap on the particle surface to form hydrophilic nanoparticles, and then reduces the surface tension of the solution [31]. The concentration of PSMA was positively correlated with the wettability of nanospheres. As an amphiphilic polymer, PSMA coated pesticide particles through hydrophobic groups. The hydrophilic groups of the PSMA were outside, expressing hydrophilic properties. Therefore, the PSMA-LC-NS with the pesticide/PSMA ratios of 1:1 exhibited good wettability.

### 2.4. Retention and Adhesion

**Figure 6:**
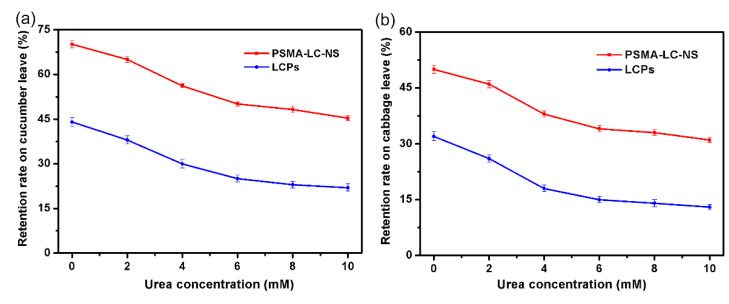
The retention rate of PSMA-LC-NS and LCPs on leaf surface after washing with different urea solutions. (**a**) *Cucumis sativus* leaf surface; (**b**) *Brassica oleracea* leaf surface.

In this study, the retention was measured to investigate the deposition and adhesion behavior of the nanospheres. As shown in Figure 6, the initial retention rates of PSMA-LC-NS and LCPs on *Cucumis sativus* leaves were 70.1% and 44% when the leaves were washed with deionized water, which suggested that PSMA-LC-NS have stronger adhesive force to crop leaf surface than that of LCPs. It may be that the surface of nanoparticles contains more hydrophilic carboxyl groups of PSMA, which can have affinity adhesion with the surface of crop leaves [32]. It was generally known that the crop leaf surface was composed of higher fatty compounds, such as fatty acids, alcohols, and aldehydes [33]. These polar groups on the crop leaf surface may interact with the surface groups of the nanoparticles [34]. Moradi et al. prepared nanoliposomes with chitosan, which can link to different molecules with negative charge, and help in crossing through nonpolar substances, such as cuticular waxes and cell membranes [35]. Yilmaz et al. constructed the microencapsulation of lambda-cyhalothrin with polyurethane-urea, which form hydrogen bonding with the water molecules, amine, hydroxyl, carboxyl, and aldehyde groups on the surface of the leaves. This interaction of hydrogen bonding improved the adhesion strength between nanoparticles and the leaf surface [36].

**Scheme 2:**
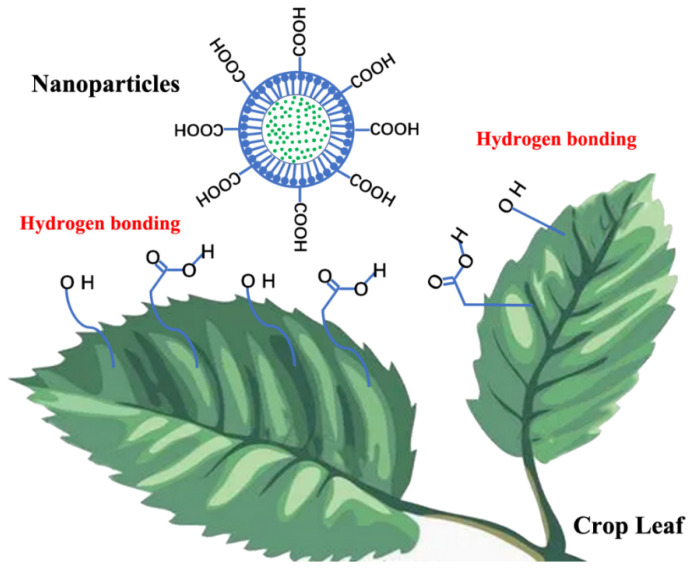
Adhesion mechanism between PSMA-LC-NS with crop leaf surface.

Urea was used as a strong hydrogen bond breaker. The adhesion mechanism between nanospheres and leaf surface was further understood by washing the blade surface with urea [37]. The concentration of urea in the flushing solution was increased in turn. When the urea concentration was 10 mM, the retention rate of PSMA-LC-NS and LCPs on *Cucumis sativus* leaves decreased to 45.3% and 22%, respectively. The results showed that the binding effect between the drug and the leaf surface was gradually weakened. The same phenomenon also occurred on the leaves of *Brassica oleracea*. The retention rate of PSMA-LC-NS on the leaves of *Brassica oleracea* reduced from 50% to 31%, and the retention rate of LCPs on the leaves of *Brassica oleracea* reduced from 32% to 13%. The results indicated that the retention rate of pesticide on crop leaves gradually decreased with the increase of urea concentration. The surface of nanoparticles contains a large number of carboxyl groups from PSMA, so the adhesive interaction between nanoparticles and crop leaf surface may be mainly realized by hydrogen bond (Scheme 2).

### 2.5. Release Behavior

**Figure 7:**
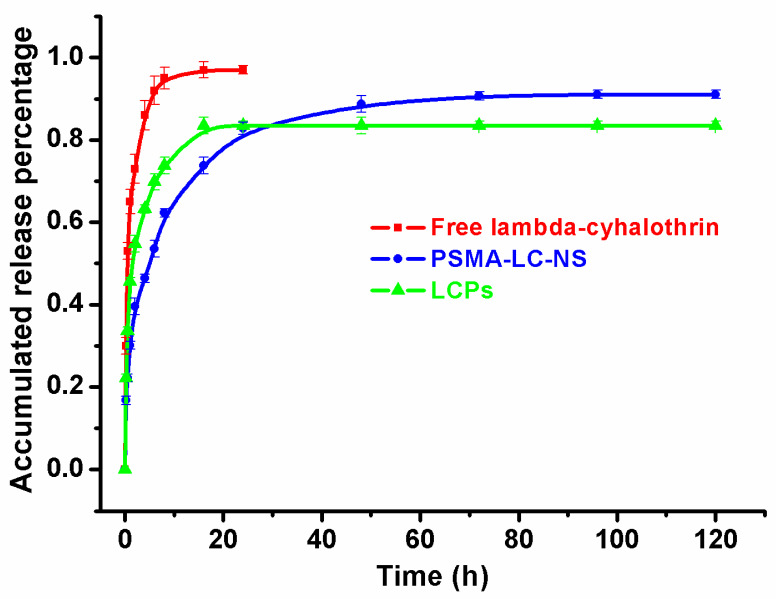
Cumulative release profiles of free lambda-cyhalothrin, LCPs, and PSMA-LC-NS.

The release profile is of importance in applying the proposed system for practical drug delivery. The lambda-cyhalothrin content of the solution at different time points was studied, and then the release performance of nanospheres was investigated. The results indicated that the prepared PSMA-LC-NS had the characteristic of sustained release compared with free lambda-cyhalothrin with the release characteristic shown in Figure 7. As known to us, the drugs loaded on nanospheres mainly existed in two forms. One part of the active component was adsorbed on the surface of the nanospheres, and the other was dispersed into nanospheres. Therefore, the release behavior is also divided into two parts. First, the drug was initially released rapidly from the surface of the nanospheres. Then, it began to release slowly from the inside of the nanospheres until the nanospheres were completely degraded after the initial burst period [38,39,40,41].

In the initial 0.5 h, the release percentages of lambda-cyhalothrin from PSMA-LC-NS and LCPs were 22.2% and 33.6%, respectively. The drug release rate of LCPs was faster than that of PSMA-LC-NS. The main reason is that the shape of LCPs is irregular, and there are many active substances adsorbed on the particle surface, so the pesticide release rate of LCPs is faster. After 16 h, the cumulative release percentages of lambda-cyhalothrin from LCPs was maintained at 83.5%. After 72 h, the cumulative release percentages of lambda-cyhalothrin from PSMA-LC-NS was 91.1%, which was more than that of the LCPs. Because PSMA-LC-NS has better encapsulation effect on the pesticide, the continuous release time is longer. This conception was confirmed by Shawer et al. The release curve of lambda-cyhalothrin chitosan nanoparticles were released explosively in the first 1 h, and then exhibited a slowly controlled release in the next 5 h [42]. In addition, the sustained release performance of PSMA-LC-NS with uniform particle size is better than that of LCPs, consistent with the previous literature [43,44,45,46]. These results indicated that PSMA-LC-NS showed good sustained release properties, raising the dissolution rate and the utilization efficiency of pesticides.

### 2.6. Stability

**Figure 8:**
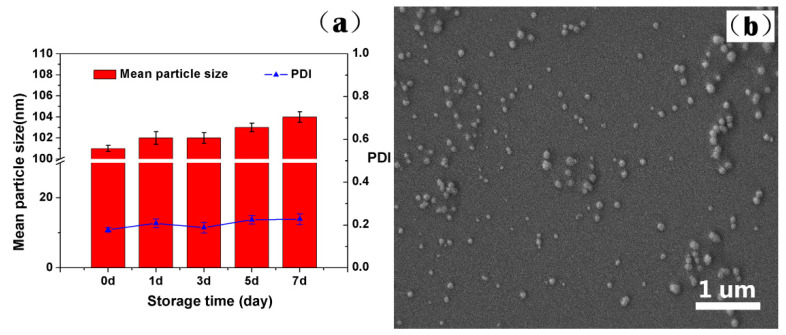
Characterization of particle size and morphology of the PSMA-LC-NS at 0 °C for 7 days. (**a**) Particle size statistical image; (**b**) SEM image.

**Figure 9:**
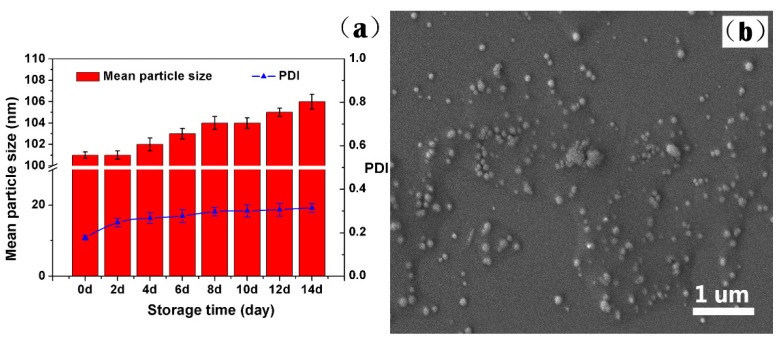
Characterization of particle size and morphology of the PSMA-LC-NS at 54 °C for 14 days. (**a**) Particle size statistical image; (**b**) SEM image.

The mean particle size and PDI were measured to evaluate the storage stability of the drug nanoparticles. In Figure 8, the mean particle size of PSMA-LC-NS increased to 104 nm in SEM image, and there was no aggregation between the particles after storage at 0 °C for 7 days. The particles have uniform distribution and approximate spherical shape. Meanwhile, the PDI maintained below 0.3, which means that the system was stable. The DLS results of the particle size of the sample stored for 7 days were consistent with that of SEM. As shown in Figure 9, the mean particle sizes of PSMA-LC-NS increased to 106 nm after storage at 54 °C for 14 days in SEM image. The particle size of the sample increased slightly at 54 °C for 14 days, but the system as a whole was stable. The results implied that the PSMA-LC-NS had good physical stability and no significant particle agglomeration.

As we know, the PDI maintained below 0.3, indicating good stability between particles [47]. The nanoparticles at different storage temperatures exhibited almost spherical morphology and relatively monodisperse distribution. The main reason may be that the particle size of nanoparticles is uniform, which reduces the aggregation from small particles to large particles, so that the change of particle size is small and the performance is stable [48,49,50]. The uniformity of particle size is positively correlated with the stability of the sample. Meanwhile, PSMA can provide negative charges for electrostatic repulsion between particles to prevent aggregation between particles. Therefore, PSMA-LC-NS had good physical stability.

### 2.7. Cytotoxicity Test

The cytotoxicity test was used to detect the effects of nanoparticles on the survival of swine testis cells after 24 h. Undiluted samples at a concentration of 500 ug/mL were added to swine testis (ST) cells; the cell survival rates after treatment with the raw lambda-cyhalothrin, LCPs, and PSMA-LC-NS were 72.4%, 89.3%, and 92.4%, respectively. The cell survival rate after treatment with PSMA-LC-NS was higher than that of raw lambda-cyhalothrin and LCPs. It can be seen that PSMA-LC-NS with microcapsule structure has a better encapsulation effect on pesticides and can delay drug release, so it has lower cytotoxicity. At the same time, we diluted the concentration of the sample to 10 ug/mL according to the field application concentration of lambda-cyhalothrin. The survival rates of ST cells after treatment with raw lambda-cyhalothrin control group, LCPs, and PSMA-LC-NS were 88.3%, 93.4%, and 97.5%. In addition, the drug carrier materials are decomposed and destroyed under the influence of the external environment during plant growth, and there is basically no drug residue after fruit ripening. Therefore, the use of PSMA-LC-NS at the recommended dose will not cause health risks.

## 3. Materials and Methods

### 3.1. Materials

Lambda-cyhalothrin (95%) was purchased from Hubei Wande Chemical Co., Ltd., Tianmen, China, poly(styrene-co-maleic anhydride) (PSMA) were obtained from Shanghai yuanye Bio-Technology Co., Ltd., Shanghai, China, urea was purchased from Sinopharm Co., Ltd., Beijing, China, and methanol was purchased from Fisher, Hampton, NH, USA. Milli-Q water (0.0667 µS/cm, total organic carbon ≤ 4 ppb) was used in all analytical experiments.

### 3.2. Methods

#### 3.2.1. Preparation of Lambda-Cyhalothrin Nanospheres Encapsulated with PSMA

Lambda-cyhalothrin nanospheres encapsulated with PSMA (PSMA-LC-NS) were prepared using the ultrasonic emulsification–solvent evaporation method. Firstly, lambda-cyhalothrin was dissolved in tetrahydrofuran fully, and PSMA were added in a certain ratio (1:1, *w*/*w*) and evenly mixed to obtain the oil phase. Then, the oil phase was injected into the water phase in a certain ratio of 1:5 for ultrasonic treatment using an ultrasonic homogenizer (JY99-IIDN, Scientz Biotechnology Ltd., Ningbo, China). The total ultrasonic time was controlled for 5 min (ultrasonic treatment for 50 s, pause for 10 s) and the ultrasonic power was 1000 W. Finally, the ultrasonic treatment solution was rotated and evaporated at 50 °C to remove tetrahydrofuran, and then lyophilized to obtain nanospheres.

The ratio of water phase to oil phase was 1:5 and the ultrasonic action time was 5 min. The lambda-cyhalothrin particles (LCPs) with the pesticide/PSMA ratios of 3:1 was selected as the control group.

#### 3.2.2. Optimization of Preparation Process

The optimization of the preparation process is very important for the size and distribution of pesticide particles. We also explored the effects of varying the preparation conditions, including PSMA content, oil phase/water phase ratio, and ultrasonic emulsification time on the particle size, morphology, and properties. In this study, particle size and polydispersity index (PDI) were used as the evaluation index to optimize the PSMA concentration, the oil phase/water phase ratio, and the ultrasonic emulsification time. First, the effect of the ratio of pesticide/PSMA on particle size was investigated by setting the ratio of 3:1, 1:1, and 1:3. Then, the effect of the ratio of oil phase/water phase on the particle size was analyzed by setting the ratio of 1:1, 1:5, and 1:10. Finally, the ultrasonic emulsification time (2, 5, and 10 min) was measured to evaluate the effect of reaction time on particle size and distribution.

#### 3.2.3. Characterization of PSMA-LC-NS

The particle sizes and morphologies of the nanospheres were characterized using scanning electron microscope (SEM, JSM-7401F, JEOL Ltd., Tokyo, Japan). First, the sample was evenly dispersed in water. A total of 3.0 µL of the solution was dripped onto the silicon wafer, then dried and sprayed with a thin layer of platinum for 40 s. The SEM images were captured under the condition of 3 kV accelerating voltage and 10 mA current. The morphology of the nanospheres was characterized using a transmission electron microscope (TEM, HT7700, Hitachi Ltd., Tokyo, Japan). The accelerating voltage was 80 kV. A total of 2 µL of diluted solution was dripped onto a carbon-coated copper grid, and then dried at room temperature. The hydrated particle sizes of the PSMA-LC-NS were examined using dynamic light scattering (DLS, Zetasizer Nano ZS90, Malvern Instruments Ltd., Malvern, UK). The polydispersity index (PDI) is used to characterize particle size distribution. It was widely known that the PDI value was less than 0.3, indicating good dispersion. Three replicates of each sample were measured.

#### 3.2.4. Wettability Test

*Cucumis sativus* as the representative of hydrophilic plants and *Brassica oleracea* as the representative of hydrophobic plants were used to characterize the wettability of the solution on the leaf surface. The contact angle of the sample solution on the leaves was measured with a contact angle apparatus (JC2000D2M, POWEREACH, Beijing, China). Firstly, the samples were dispersed and dissolved with deionized water, then the plant leaves were fixed, and 5 µL of aqueous solution (0.05%, *w*/*w*) was dropped onto the leaves through the contact angle apparatus, and then the droplet was captured in real-time. The contact angles were measured using the five-point fitting analysis method. The final values were the average of three measurements. Each concentration corresponds to three parallel experimental groups.

#### 3.2.5. Retention Test

PSMA-LC-NS and LCPs were diluted to the same concentration containing the same amount of lambda-cyhalothrin, and then were sprayed onto clean and fresh *Cucumis sativus* and *Brassica oleracea* leaves, respectively. The treated leaves were dried at room temperature. A urea solution was used as a flushing agent to explore the interaction mechanism between pesticide molecules and crop leaves. Half of each leaf was washed with 100 mL urea solution, and the other half of each leaf was protected from washing with the urea solution. The concentration gradient of urea solution was 0, 2, 4, 6, 8, and 10 mM. Lambda-cyhalothrin in urea-treated and urea-untreated leaves was extracted and determined, respectively. The retention of pesticide in leaves was determined by high performance liquid chromatography (HPLC, Agilent1260, Agilent Ltd., Santa Clara, CA, USA). Each experiment was repeated three times.

The lambda-cyhalothrin concentrations were analyzed using HPLC. The parameters were as follows: C18 column (5 um × 4.6 mm × 150 mm); ultraviolet detector (UV, 245 nm); mobile phase (acetonitrile and water, *v*/*v* = 85:15); flow rate (1.0 mL/min); and injection volume (20 uL).

#### 3.2.6. In Vitro Release

Raw lambda-cyhalothrin, LCPs, and PSMA-LC-NS were dissolved in 5 mL methanol, respectively. The treated dialysis bag was filled with the above solution and then suspended in a 250 mL brown reaction bottle containing 95 mL of 60% methanol solution. It was placed in a 25 °C constant temperature shaking table for oscillatory dialysis (100 r/min). A total of 5 mL of dialysate was taken from the reaction bottle at 0.2, 0.5, 1, 2, 4, 6, 8, 16, 24, 48, 72, 96, and 120 h, respectively. The release medium with equal volume was supplemented into the reaction bottle to keep the total volume of 100 mL in the reaction bottle. The cumulative release of lambda-cyhalothrin was calculated in the sustained-release solution at different times according to the standard curve. The assay was performed 3 times for each sample.

The standard curve of lambda-cyhalothrin was prepared as follows: 10 mg of the standard sample of lambda-cyhalothrin was dissolved in a 50 mL volumetric flask with methanol. Then, the mother liquor was diluted successively to obtain standard solutions of 200, 100, 50, 20, 10, and 5 ug/mL. Finally, the samples were injected according to the conditions of HPLC, and the standard curve was calculated according to the solution concentration and peak area.

#### 3.2.7. Stability

The stability of PSMA-LC-NS at different storage temperatures was evaluated. The particle size and PDI are the main indexes to measure the stability of nanospheres during the whole storage process. The freeze-dried solid samples were stored at 0 °C for 7 days and at 54 °C for 14 days to explore the physicochemical stability. The stored samples were diluted to 0.5% (*w*/*w*) to determine the hydrated particle size at a specific time. Moreover, the particle size and morphology of the nanospheres were also characterized using SEM. The stability of the nanosphere system was evaluated according to the particle size and agglomeration state.

#### 3.2.8. Cytotoxicity Test

Swine testis (ST) cells were used to detect the cytotoxicity of PSMA-LC-NS. The cells were subcultured and tested for cytotoxicity with the 3-(4, 5- dimethylthiazol-2-yl)-2,5-diphenyltetrazolium bromide (MTT) assay. Swine testis cells in the logarithmic growth stage were digested with 0.25% trypsin to make a single cell suspension, and the cell suspension was inoculated into 96-well plates according to 2 × 10^4^ cells / well. After 24 h of culture in the medium, the culture medium was removed, and raw lambda-cyhalothrin, LCPs, and PSMA-LC-NS diluents were added for 24 h. The initial concentration of the lambda-cyhalothrin was 500 ug/mL, and the concentration of the sample after dilution was 10 ug/mL. Untreated ST cells were used as a control. After the samples were cultured at 37 °C and 5% CO_2_ for 24 h, and were cultured for another 3 h in a culture medium containing MTT (0.5%), then the medium was carefully removed. Dimethyl sulfoxide (150 µL/well) was added, and then the solution was gently shaken and mixed. Finally, the absorbance of the solution at 490 nm was measured with a microplate reader. The cell viability was expressed as [OD490(sample)/OD490(control)] × 100.

#### 3.2.9. Statistical Analysis

The statistical data were performed by IBM SPSS Statistics 21.0, and was presented as mean ± standard deviation (SD). Least significant difference (LSD) was used to analyze the data. A probability (*p*) of less than 0.05 means significant differences.

## 4. Conclusions

In summary, we successfully established an innovative and facile method for insoluble pesticides. The hydrophilic lambda-cyhalothrin nanospheres with PSMA as encapsulation material were developed via the ultrasonic emulsification–solvent evaporation method, which exhibited excellent monodispersion and uniformity. The effects of the PSMA concentration, oil/water ratio, and phacoemulsification time on the particle size and morphology of the lambda-cyhalothrin nanoparticles were inspected to determine the process conditions. The optimal preparation conditions for PSMA-LC-NS were as follows: the ratio of pesticide/PSMA was 1:1; the volume ratio of the oil phase/water phase was 1:5; and the ultrasonic action time was 5 min.

The mean particle size of the prepared PSMA-LC-NS under the above conditions was approximately 103 nm, and showed a uniform size without aggregation, smooth surfaces without cracks, and good dispersion. In addition, PSMA-LC-NS with small particle size had good dispersibility and wettability in an aqueous solution due to the larger specific surface area and the faster dissolution rate of the active ingredient. In addition, PSMA-LC-NS have a stronger adhesive interaction with the crop leaf surface and exhibited excellent continuous sustained release due to the coating effect of PSMA. PSMA-LC-NS with uniform particle size distribution have good stability and have a small particle aggregation effect. Meanwhile, the cytotoxicity test showed that the use of PSMA-LC-NS at the recommended dose did not cause health risks. In future work, the same method would be applied to encapsulate hydrophobic pesticides, and extended for exploring nanoparticles with different functional agents.

## Figures and Tables

**Table 1:** Mean particle size and zeta potential of the nanospheres.

Samples	Mean Particle Size (nm)	Zeta Potential (mV)
PSMA-LC-NS	105 ± 0.6	−31.5
lambda-cyhalothrin particles	248 ± 5.6	−16.5
